# RandAL: a randomized approach to aligning DNA sequences to reference genomes

**DOI:** 10.1186/1471-2164-15-S5-S2

**Published:** 2014-07-14

**Authors:** Nam S Vo, Quang Tran, Nobal Niraula, Vinhthuy Phan

**Affiliations:** 1 University of Memphis, Memphis, TN 38152, USA

**Keywords:** next-gen sequencing, short read alignment, randomization

## Abstract

**Background:**

The alignment of short reads generated by next-generation sequencers to genomes is an important problem in many biomedical and bioinformatics applications. Although many proposed methods work very well on narrow ranges of read lengths, they tend to suffer in performance and alignment quality for reads outside of these ranges.

**Results:**

We introduce RandAL, a novel method that aligns DNA sequences to reference genomes. Our approach utilizes two FM indices to facilitate efficient bidirectional searching, a pruning heuristic to speed up the computing of edit distances, and most importantly, a randomized strategy that enables effective estimation of key parameters. Extensive comparisons showed that RandAL outperformed popular aligners in most instances and was unique in its consistent and accurate performance over a wide range of read lengths and error rates. The software package is publicly available at https://github.com/namsyvo/RandAL.

**Conclusions:**

RandAL promises to align effectively and accurately short reads that come from a variety of technologies with different read lengths and rates of sequencing error.

## Background

The alignment of reads to genomes is an important problem in many biomedical applications that relied on next-generation sequencing technologies. This problem is motivated by the fact that genomes for many species have been sequenced. And since one expects genomes within the same species differ little, such "referenced" genomes can facilitate the assembly of new genomes of other individuals within the same species from short reads. To address this problem, researchers have proposed many approaches together with software packages. Nevertheless, sequencing technologies have advanced rapidly, rendering many of these approaches ineffective or inefficient or both. One aspect that continually changes is the read length. Advanced technologies generally produce longer reads (with better accuracy). On the other hand, technologies that produce shorter reads can be less expensive and are therefore attractive in terms of cost. Thus, it is desirable to have algorithms and tools that perform well across different read lengths ranging from 35 to several hundreds basepairs.

Nevertheless, many existing algorithms struggle to perform consistently across a wide range of read lengths. Methods such as Bowtie [1] and Burrows-Wheeler Alignment (BWA) [2] tend to perform better with shorter reads. Bowtie uses the Burrows-Wheeler Transform (BWT) and FM index to build a permanent index of the reference genome. It then applies backtracking algorithm to find alignments. BWA also utilizes the BWT, but unlike Bowtie, can handle gaps and mismatches in the reads. More advanced versions of these methods include Bowtie2 [3] and BWASW [4] which are designed to work with longer reads. Bowtie2 can align reads with gaps and works better than Bowtie at longer reads. BWA-SW exploits the BWT and several heuristics to speed up the local alignment of reads.

Many techniques utilize data structures and techniques such as the BWT, FM index, suffix arrays, suffix trees/tries, hash tables or q-grams [5-11], aiming to speed up substring querrying. Additional heuristics are also used to enhance efficiency. Bowtie2 [3] and CUSHAW2 [12], for example, use *seeds *to quickly identify true candidates for alignment. GASSST [9] uses a filtering technique to reduce noisy seeds. Implementations of some of these approaches, e.g. Bowtie2, CUSHAW2, take advantages of parallelism or special-purpose architectures. The use of heuristics can improve performance several folds, but might lead to over-tuning parameters to a particular set of inputs, e.g. read lengths, species, or base error rates.

We introduce RandAL, an aligner based on a novel algorithm that performs consistently well over a wide range of read lengths, from 35 to several hundreds base pairs. We employ two FM indices for efficient bidirectional (exact) substring matching. To deal with inexact matching (i.e. allowing gaps), first, we find common substrings between reads and the reference genome. Then, these common substrings are extended to complete alignments based on a bounded threshold on the edit distance. We use a special pruning mechanism to shorten vastly the running time of computing edit distances in a vast majority of cases. The use of randomization in aligning reads to genomes increases the probability of finding seeds quickly and enables us determine methodologically important parameters to speed up the entire alignment process. Preliminary results show that our algorithm performed consistently well on a wide range of read lengths across several bacterial and eukaryotic genomes. The alignment quality of our method was better or generally as good as that of all compared methods.

## Methods

Given a reference genome  S and a set of reads **R **= {*r*_1_, …, *r_n_*}, the main problem is to align each *r_i _*to  S. The reference genome  S and the reads are DNA sequences, or strings over the alphabet of 4 characters, Σ = {*A, G, C, T*}. The alignment of a read *r *to  S is essentially finding a substring of  S that matches *r *the most. At the moment, we assume that these reads are not *paired-end *reads. The set of reads **R **are substrings of another genome  R that is different from, but belongs to the same species as  S. By aligning reads in **R **to  S, we implicitly reconstruct the genome  R.

Our strategy for read alignment is based on these ideas:

1 Detection of identical substring matches between *r *and  S is based on common substrings of *r *and  S. As we know *r *and  S differ only slightly, we expect long common substrings exist.

2 A special data structure called the FM index is used to facilitate memory-efficient, time-optimal exact string matching. This data structure facilitates efficient detection of long common substrings between *r *and  S.

3 Randomization is employed to find common substrings between *r *and  S efficiently and *methodologically*. Randomization empowers us to methodologically determine important parameters that are used in critical steps of the algorithm. This translates into consistent performance in terms of time and accuracy across different species.

4 To account for insertion/deletion polymorphisms, we utilize the edit distance to provide an accurate measure for read alignment. Additionally, we employ a pruning heuristic to shorten the computation of edit distance, without com promising quality of alignment.

These ideas will be discussed in greater detail in the following sections.

### Indexing the reference genome

Naive string matching takes quadratic time and therefore is too costly. Researchers have used data structures such as suffix tree, suffix array, and FM index to speed up string matching significantly. The FM index [13] in particular is desirable because it allows exact string matching to be done optimally in *O*(*m*) time, where m is the length of the query (i.e. the read), and is very space efficient. The FM index of the genome is a substring index that takes advantage of properties of the Burrows-Wheeler transform to search incrementally all suffices of a read in the reference genome. This allows linear time (in read length) searching for exact substring matches. By design, the search direction is in reverse (backward) order with respect to the sequence.

To facilitate bidirectional string matching (to be discussed next), we employ two *FM indices*. A conventional FM index that traces substring matches *backward *is denoted as  Ɓ. To facilitate searching in the forward dimension, we created an FM index for the reverse of the reference genome,  S. Searching using this index, denoted as  Ƒ, is equivalent to search in the forward direction in  S. The pair of indices (Ƒ,Ɓ) helps us identify long identical stretches of DNA in the reference genome  S and each read *r_i_*.

### Finding common substrings between reads and genomes

**Figure 1:**
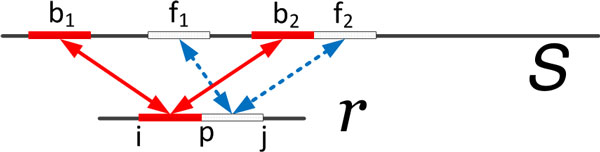
**Illustration of Algorithm 1: finding common substring**. *r*_*i…p−*1 _and *r*_*p…j *_may match several substrings of the genome  S, but fews of these (e.g. *b*_2 _and *f*_2_) form contiguous substrings.

Given a read *r *and a specific position *p *in *r*, Algorithm 1 outlines the steps in finding longest common substrings of *r *and the reference genome  S, *with respect to p*; Figure 1 illustrates the conceptual goal of this algorithm. Longest common substrings (with respect to *p*) are constructed by concatenating maximal matches between substrings of  S and those of *r*, which begin and end at *p*. Searching for matches between substrings of  S and substrings of *r *is facilitated by the backward and forward FM indices  Ɓ and  Ƒ. To save time and reduce false positives, we only consider common substrings with lengths at least *W*.

The choice of *W *is important. If *W *is too small, *M *is large, and we will consider many common substrings between the read and the genome to construct alignments between the read and the genome. The more common substrings we consider, the more likely we can find the correct position of the read in the genome to align; but we also more likely make mistakes of aligning the read to an incorrect position. In other words, with smaller *W*, we might get more true positives (correct alignments) and more false positives (incorrect alignments) at the same time. On the other hand, if *W *is too large, we might not be able to find any common substrings and consequently unable to align the read to the genome. Therefore, inappropriate choices of *W *results in bad performance.

### Algorithm 1 CommonSubstrings(read *r*, position *p*)

1: Let *B *be substrings of reference genome  S, which match exactly & maximally to *r*_*i...p*-1_.

2: Let *F *be substrings of reference genome  S, which match exactly & maximally to *r*_*p...j*_.

3: *M *:= ∅

4: **for **each *b *∈ *B ***do**

5:    **for **each *f *∈ *F ***do**

6:        Let *s *:= *b *⊕ *f *be a concatenation of *b *and *f*.

7:        **if ***s *is a contiguous block in  S and *|s| ≥ W ***then**

8:            *M *:= *M *∪ *s*

9: **return ***M*

Our strategy for determining good values of *W *is based on randomization. As we shall see soon, the value *p *given to Algorithm 1 would be a random index of the read. To calculate *W*, first suppose that the correct substring of the reference genome  S to align to the read *r *is *r'*. Let *d *be the edit distance between *r *and *r'*. These *d *mismatches divide *r *into *d *+ 1 blocks. Each block (except the last one) includes the closest mismatch to it. Let the sizes of the blocks be *m*_1_, *m*_2_, … … …, *m*_d+1_. We have $|r|\;=m=\sum_{i=1}^{d+1}m_{i}$.

The random choice of *p *implies that the common substring found by Algorithm 1 would be a random block, which is selected with probability $p_{i}=\frac{m_{i}}{m}$. This implies that the expected size of block *i *is $E\left[S_{i}\right]=m_{i}p_{i}=\frac{m_{i}^{2}}{m}$. Thus, the expected size of a random block, i.e. the expected length of the common substring, is $E\left[X\right]=\sum_{i=1}^{d+1}E\left[X_{i}\right]=\sum_{i=1}^{d+1}\frac{m_{i}^{2}}{m}$.

The Cauche-Schwarz inequality tells us that

$${\left(\overset{d+1}{\underset{i=1}{\sum }}\frac{1}{d+1}\frac{m_{i}}{m}\right)}^{2}\leq \overset{d+1}{\underset{i=1}{\sum }}{\left(\frac{1}{d+1}\right)}^{2}\overset{d+1}{\underset{i=1}{\sum }}{\left(\frac{m_{i}}{m}\right)}^{2}$$

After simplifying, these imply that $E\left[S\right]\geq \frac{m}{d+1}$. In other words, we have established that:

**Lemma: **The expected length of the common substring between a read and the reference genome found by Algorithm 1 is at least $\frac{m}{d+1}$.

Although we do not know what *d*, the distance between *r *and its aligned substring *r'*, is, it can be estimated by the rates of single nucleotide polymorphism (SNP) of the given genome and given rate of sequencing error. Let *b *be the rate of each nucleotide being mutated or sequenced erroneously, which we may assume to be distributed by a binomial distribution with mean *µ *= *mb *and variance *σ*^2 ^= *mb*(1 *- b*), where *m *is the read length.

Although we do not know exactly what *d *is, its upper bound *t *might be estimated by *µ *+ *cσ*, for some constant *c*. With 100,000 reads, we found that *c *= 4 produces good performance with high true positives and low false positives.

In summary, the two critical parameters of our method *t *and *W *are methodologically derived as follows:

• The upper bound of the distance between a read and its aligned string, $t=\left\lceil mb+4\sqrt{mb\left(1-b\right)}\right\rceil .$

• The lower bound of the expected length of common substrings, $W\sim \frac{m}{t}\leq \frac{m}{d+1}\leq E\left[S\right]$.

*W *appears in Algorithm 1, and *t *appears in Algorithm 2, which is the next step after finding common substrings between reads and the reference genome.

**Algorithm 2 **AlignRead(read *r*)

1: *p *:= 1

2: *m *:= *|r|*

3: **for ***i *from 1 to *A ***do**

4:    *C *:= ∅

5:    *M *:= CommonSubstrings(*r, p*)

6:    **for **each *s *∈ *M *, which is a substring of  S**do**

7:        Let *r_i…j _*be the substring of *r *that matches *s *exactly.

8:        Let *s_L _*be the (*i − *1)-substring of  S, preceding *s*

9:        Let *sR *be the (*m − j*)-substring of  S, following *s*

10:        *d *:= edit-dist(*r*_1*…i−*1_, *s_L_*) + edit-dist(*r*_*j*+1*…m*_, *s_R_*)

11:        **if ***d ≤ t ***then**

12:            *C *:= *C *∪ (*s_L _*⊕ *s *⊕ *s_R_*)

13:    **if ***C *has at least one sequences **then**

14:        Return "fail to align", if *C *has more than 2 sequences.

15:        Otherwise, align read *r *to each sequence of *C*. STOP.

16:    *p *:= random(1,*|r|*)

17: **return **"fail to align"

### Extending common substrings to align reads to referenced genomes

Using long exact common substrings as seeds to align reads to genomes is similar to [3,12]. Our approach promises to be efficient because instead of exhaustively traversing indices of a read to find optimal common substrings, we find common substrings with respect to random index *p *of the read.

**Figure 2:**
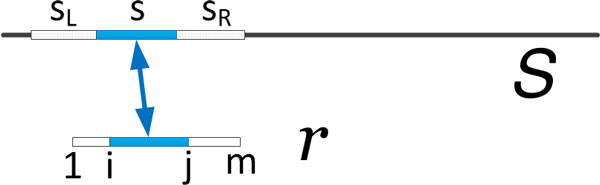
**Illustration of Algorithm 2: extending common substring to alignment**. Alignment of a read *r *to the reference genome  S by extending a common substring of *r *and  S (found in Algorithm 1). There are generally many substrings of  S that match identically to the substring *r_i…j _*of *r*.

In Algorithm 2, we iterate at most *A *times to find long common substrings between  S and each read *r*. In each iteration, given a random position *p*, we invoke Algorithm 1 to find the longest common substrings (*M*) of  S that match to a substring of *r *with respect to *p*. As illustrated in Figure 2, each string *s *∈ *M *corresponds exactly to a substring *r_i…j _*of *r*. This exact match between *r_i…j _*and *s*, naturally, pairs up *r*_1*…i−*1 _(a prefix of *r*) to *s_L_*, the corresponding substring of  S preceding *s*, and *r*_*j*+1*…m *_(a suffix of *r*) to *s_R_*, the corresponding substring of  S following *s*. If the total edit distances of these two pairs are less than the previously calculated upper-bound *t*, we align *r *to the corresponding substring of  S.

Note that in the first iteration, the position *p *is 1 and not a random index of *r*. The reason for this is that we would like the method of finding long common substrings (Algorithm 1) to be *symmetrical *in the sense that *b *and *f *could "wrap around" *r*. In other words, when *p *= 1, *b *is a suffix of *r *and *f *is a prefix of *r*. In this case, the concatenation of *b *⊕ *f *is not a contiguous substring, but rather two contiguous strings separated by a big gap. This conceptualization of "wrapping around" the read, or thinking of it as a circular instead of linear string, turns out to be quite effective in practice. In many cases, *p *= 1 leads to very long common substrings that lead to correct alignments of reads.

If we cannot align *r *to any substring of  S after *A *attempts, then *r *is unaligned to  S, the reference genome. So, it is important to choose *A *appropriately. If *A *is too small, there will be many unaligned reads. If *A *is too large, the algorithm is slow. To select an appropriate value of *A*, let us again assume that the read and its correct alignment to the genome differ in *d *places (again *d ≤ t*), consequently diving the reads into *d *+ 1 blocks. We want to select a value for *A *so that the longest block (longest common substring) can be sampled with high certainty. The probability that the longest block is selected (i.e. if a random index *p *lands inside it) is $\frac{m^{*}}{m}$, where *m*^∗ ^is the length of the longest block. On the other hand, the Pigeonhole Principle dictates that $m^{*}\geq \frac{m}{d+1}$ (Otherwise, the total lengths of *d *+ 1 blocks would be less than *m*.) This means, $d+1\geq \frac{m}{m^{*}}$, which is the expected number of iterations to sample *p *to select the longest block.

Thus, setting *A *= *t *+ 1 *≥ d *+ 1, the longest common substring between a read and the genome is sampled expectedly after *A *iterations. Further, if *A *= *c … *(*t *+ 1), then the probability of landing in the longest block is exponentially increased as a function of *c*. Trading for speed, *c *= 1 seems to work fine in practice, because even if Algorithm 1 does not return the longest common substring, it is often possible to extend it to find the correct alignment for the read. But longest common substrings minimizes the chance of running into repeats in the genome; i.e. common substrings upon which extensions will lead to incorrect alignments.

### Fast heuristic for computing edit distances

Computing edit distances consumes much time of the alignment algorithm (Algorithm 2). In steps 10-11 of Algorithm 2, we compute the edit distance between a read and a substring of the genome and discard it if the distance is greater than *t*. As each read often match with few substrings of the genome, we expect that such edit distances often exceed *t*. Examining lines 10-11 of Algorithm 2, we see that actually we do not need to compute the exact value of *d*(*x, y*), the edit distance of *x *and *y*, as long as we can answer correctly the query *d*(*x, y*) *≤ t*.

We claim that the edit distance of *x *and *y, d*(*x, y*) *≤ t *if and only if Bound(*x, y, t*) *≤ t*, where Bound is defined in Algorithm 3. To see this, observe that

• If *d*(*x, y*) *≤ t*, then Bound(*x, y, t*) returns *d*(*x, y*).

• If *d*(*x, y*) *> t*, then Bound(*x, y, t*) returns either *d*(*x, y*) or *t *+ 1. The only difference between Bound and the conventional edit distance lies in line 6 of Algorithm 3. Analyzing line 5, we see that once *d_i,j _> t *for 1 *≤ j ≤ m *(line 6), then *d_m,m _> t*.

If *d*(*x, y*) *> t*, Bound(*x, y, t*) might not compute the edit distance correctly. Nevertheless, *d*(*x, y*) *≤ t *if and only if Bound(*x, y, t*) *≤ t*. For aligning reads to bacterial genomes, Bound is much faster than the worst-case complexity Θ(*m*^2^).

**Algorithm 3 **Bound(*x, y, t*)

1: *d_i,0 _*:= 0 for 0 *≤ i ≤ |x|*

2: *d*_0*,j *_:= 0 for 0 *≤ j ≤ |y|*

3: **for ***i *:= 0 to *|x| ***do**

4:    **for ***j *:= 1 to *|y| ***do**

5:        *d_i,j _*:= min(*d*_*i−*1*,j−*1_+(*xi *== *y_j_*), *d*_*i−*1*,j*_+ 1, *d*_*i,j−*1_+1)

6:    **return ***t *+ 1 if *d_i,j _> t *for 1 *≤ j ≤ *max{*|x|, |y|*}

7: **return ***d_|x|,|y|_*

## Results

RandAL is implemented in C++; FM-index codes are adapted from an external library (http://code.google.com/p/fmindex-plus-plus). We compared our method against several aligners including Bowtie [1], BWA [2], Bowtie2 [3], BWA-SW [4], and CUSHAW2 [12]. We chose these methods based on the fact that they are recently published, very popular and their software are available. Comparison tests were conducted on a workstation with two Intel Xeon E5-2680 2.70GHz CPU and 64 GB RAM.

Each aligner is tested with 100,000 simulated reads generated for each of 6 bacterial genomes and 6 chromosomes of eukaryotic genomes. Sizes of these genomes range from 1.3 and 28 millions bases; see Table 1. Genomes were obtained from EMBL-EBI (http://www.ebi.ac.uk/genomes). Since recent sequencing technologies produce read lengths ranging from 35 to 400bp at greater speed and lower cost than previous technologies (e.g. Sanger sequencing) [14], we choose this range of read lengths to evaluate the methods. More specifically, the reads were generated at lengths 35, 51, 76, 100, 200, and 400 as these lengths have been mentioned in the literatures. The *wgsim *C program, part of the SAMtool package [15], was used to generate reads.

**Table 1:** Reference genomes, obtained from EMBL-EBI (http://www.ebi.ac.uk/genomes).

	Genome	Accession #	Size (bp)
Bacteria	*Wolbachia endosymbiont of Drosophila melanogaster*	AE017196	1,267,782
	*Staphylococcus aureus subsp. aureus TW20*	FN433596	3,043,210
	*Escherichia coli 042*	FN554766	5,241,977
	*Pseudomonas aeruginosa LESB58*	FM209186	6,601,757
	*Streptomyces hygroscopicus subsp. jinggangensis 5008*	CP003275	10,145,833
	*Sorangium cellulosum So ce56*	AM746676	13,033,779

Eukaryota	*Debaryomyces hansenii CBS767 chromosome A*	CR382133	1,249,940
	*Ectocarpus siliculosus strain Ec 32 chromosome LG01*	FN649726	3,745,584
	*Schizosaccharomyces pombe chromosome I*	CU329670	5,579,133
	*Caenorhabditis elegans chromosome I*	BX284601	15,072,434
	*Taeniopygia guttata chromosome 10*	CM000527	20,806,668
	*Drosophila melanogaster chromosome 3R*	AE14297	27,905,053

Extensive comparisons were performed using SAMtool's default settings, with base error rate at 2%; 15% of polymorphisms are indels with lengths drawn from a geometric distribution with density 0.7 *∗ *0.3*l−*1. Additionally, we present summary results for 1% and 4% base error rates with similar trends and conclusions.

Aligned reads from aligners are evaluated using the *wgsim_eval *Perl script, a part of the SAMtool package, using the default setting in which a read is mapped correctly if its mapping position is within a distance of 20 from the correct position. To compare alignment quality, we defined:

$$\begin{array}{c} Precision=\frac{\#\text{ correctly aligned reads}}{\#\text{ aligned reads}}\\ Recall=\frac{\#\text{ correctly aligned reads}}{\#\text{ reads}} \end{array}$$

### Alignment quality of 6 aligners

**Figure 3:**
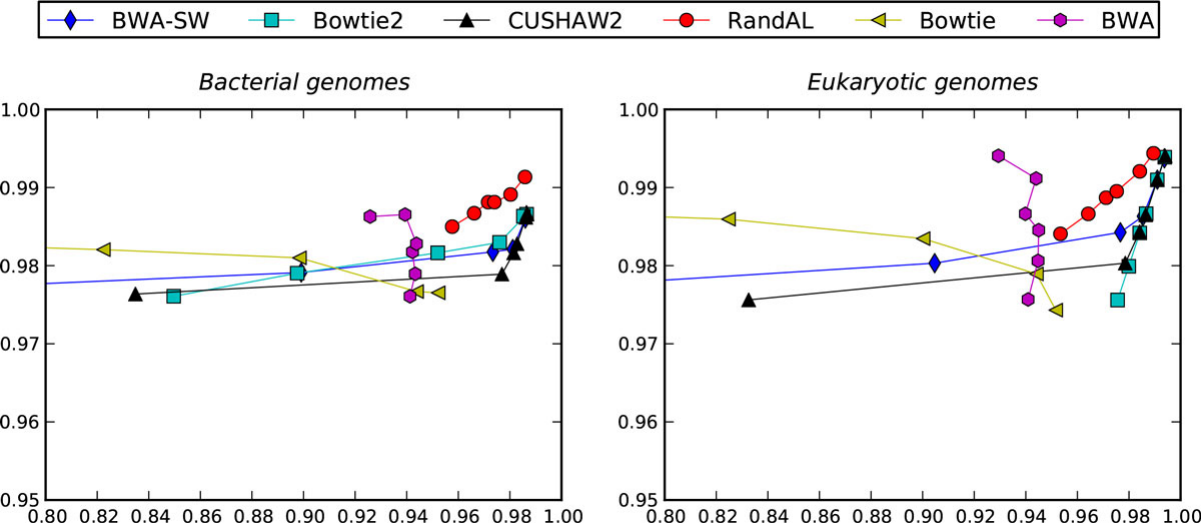
**Alignment performance across 6 different read lengths**. Recall (x-axis) versus Precision (y-axis) averaged across bacterial genomes and eukaryotic genomes, respectively, at read lengths of 35 bp, 51 bp, 76 bp, 100 bp, 200 bp and 400 bp.

At the outset, we found that Bowtie and BWA were decent performers when read lengths were short, i.e. between 31-56 bases. When read length increased, however, these two aligners were not competitive. Figure 3 shows the average performance (*precision *in the y-axis versus *recall *in the x-axis) of 6 aligners on 6 bacterial genomes and 6 eukaryotic genomes, respectively. Both BWA and Bowtie suffered from a decrease of precision as read length increases. For BWA, recall peaked at around 94% even at longer reads. Bowtie did better at recall for longer reads than BWA, but it was still not competitive to the other 4 aligners, including RandAL. Its bad performance at longer reads is unacceptable because technological trends tend to produce longer reads. For this reason, we will drop them out of head-to-head comparisons at this point, and will only compare the 4 best aligners: Bowtie2, BWA-SW, CUSHAW2 and RandAL.

A closer look at Figure 3 reveals that BWA-SW was relatively competitive but come roughly in the last place. There is no consistent winner (in terms of both precision and recall) among the top 3 performers, Bowtie2, CUSHAW2, and RandAL. Nevertheless, we can see that RandAL did noticeably better in terms of precision and was still competitive in terms of recall. Importantly, we see that across the wide range of read lengths from 35 to 400 for both bacterial and eukaryotic genomes, the performance of RandAL was consistently high in terms of both precision and recall; average precision was never below 0.98 and average recall was never below 0.95. This consistency distinguishes RandAL from the other top aligners.

**Figure 4:**
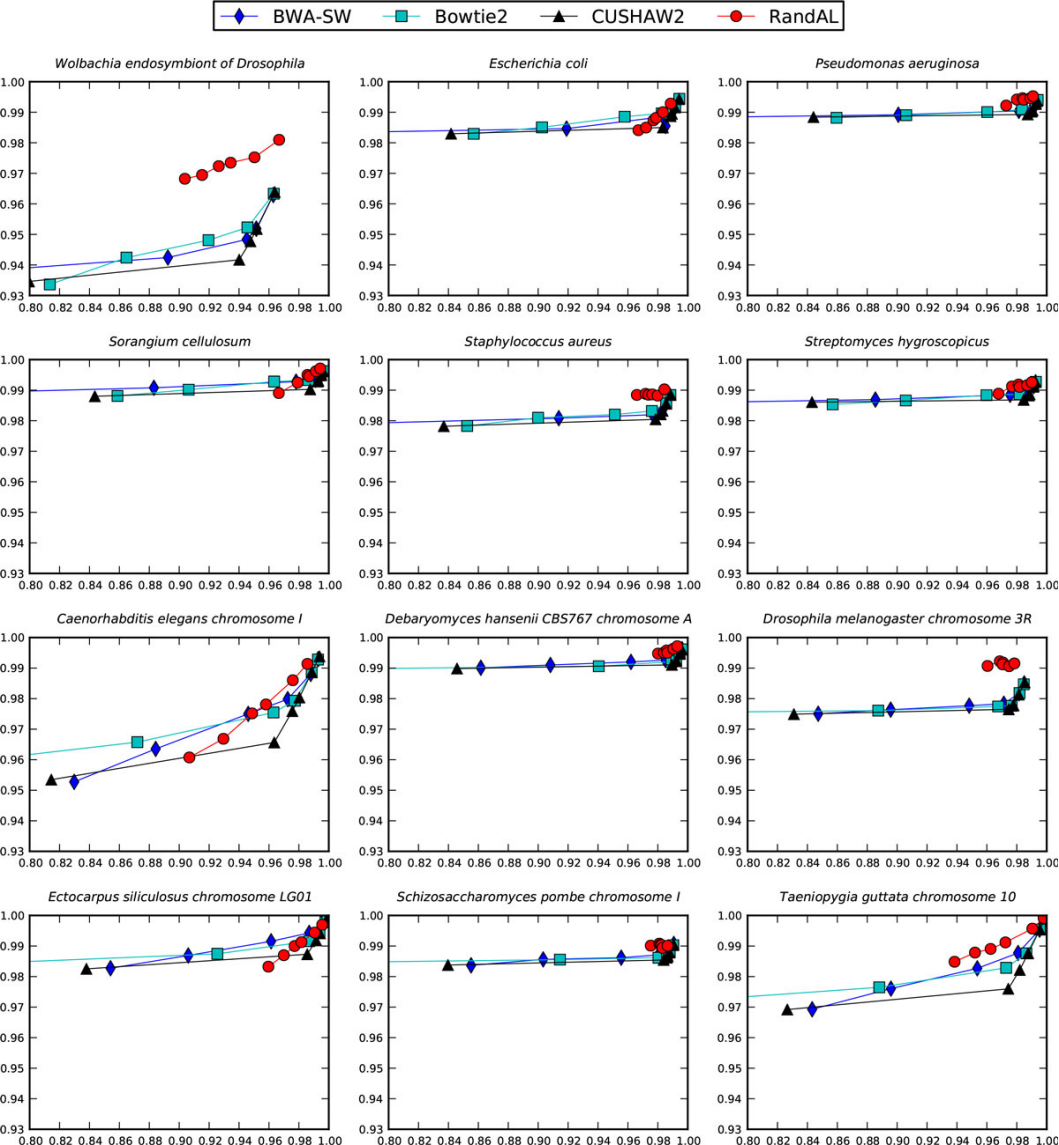
**Recall versus precision of top 4 aligners**. Performance of top aligners on bacterial genomes (top 6 figures) and eukaryotic genomes (bottom 6 figures). X-axis is recall; Y-axis is precision.

An even closer look at individual bacterial and eukaryotic genomes (Figure 4) further reinforces the consistency in performance of RandAL. The lowest precision RandAL got in all 12 genomes was about 0.96, and the lowest recall was about 0.90. In comparison, for the other top performers, the lowest precision was about 0.93 and lowest recall was about 0.80.

All top 4 aligners perform really well in both precision and recall as read length increases. Their performance was quite similar at 400 read length. At shorter read lengths, however, RandAL outperformed the rest, often in both precision and recall.

### Rates of misalignment of top 4 aligners

Misalignment means aligning a read at an incorrect position. Misalignment increases the likelihood of running into problems later when we are interested in assembling reads into a complete genome and to identify where the constructed genome different from the reference genome (SNP calling).

**Figure 5:**
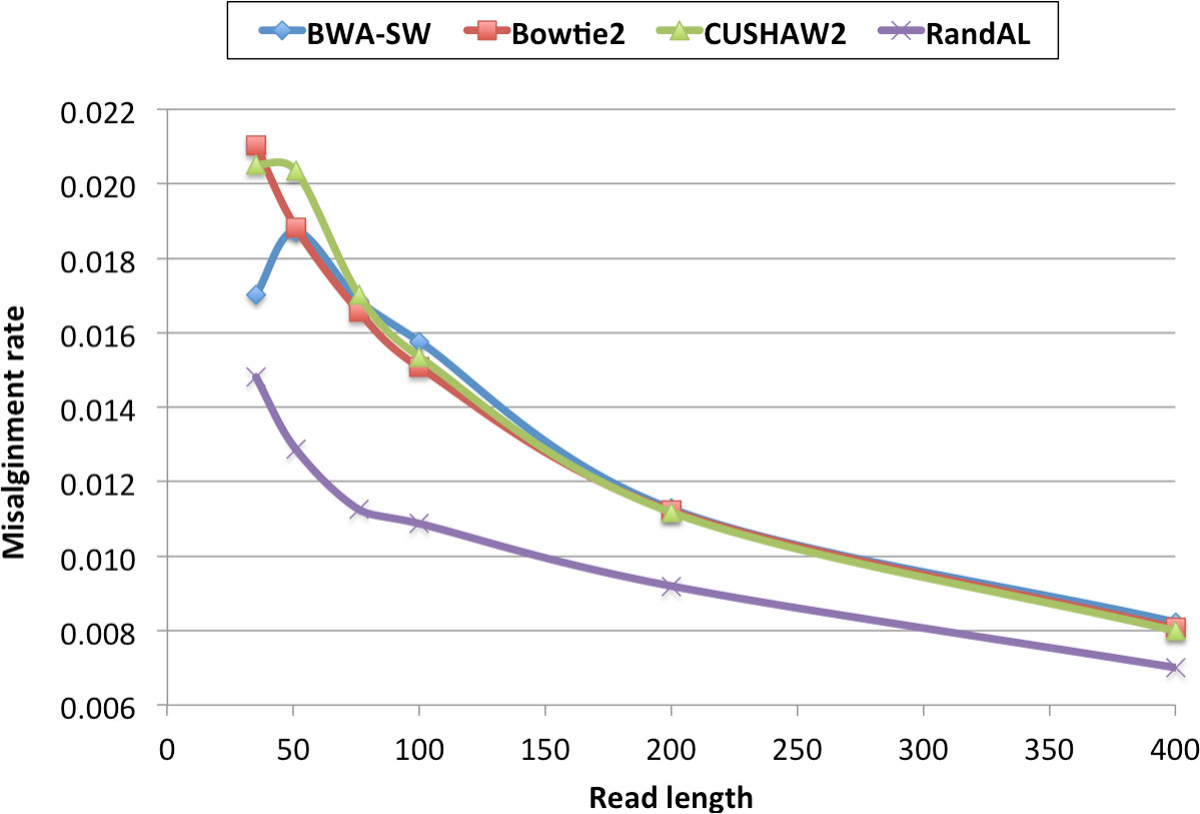
**Rate of misalignment**. Rate of misalignment averaged across bacterial and eukaryotic genomes.

The misalignment rate is calculated by dividing the number of incorrect aligned reads by the total number of reads. Figure 5 shows that averaging across all bacterial and eukaryotic genomes, RandAL got noticeably lower misalignments than the other aligners at all different read lengths. This result suggests that RandAL will likely work well with other tools and methods that require alignment of reads to reference genomes.

### Alignment quality at different base error rates

We have compared performance of 4 different methods using a base error rate of 2%; each nucleotide is mutated with the probability of 2%. Due to the lack of space, we cannot present a comprehensive comparison at different base error rates, as we have at 2%. Nevertheless, analyses at different base error rates show similar behaviors as we have observed at 2% error rates. We present a summary analysis at two other base error rates of 1% and 4% at read lengths of 35 bp, 100 bp, and 400 bp. Table 2 summarizes the average precision and recall of the top 4 aligners. These numbers suggest the followings:

**Table 2:** Average precision and recall at 1% and 4% base error rates.

		35 bp		100 bp		400 bp	
		
		Precision	Recall	Precision	Recall	Precision	Recall
1% base error	BWA-SW	97.60	82.86	98.30	98.29	98.98	98.98
	Bowtie2	97.60	93.40	98.31	98.25	99.00	**99.00**
	CUSHAW2	97.59	92.81	98.33	**98.33**	98.99	98.99
	RandAL	**98.88**	**95.49**	**99.09**	97.04	**99.18**	98.45

4% base error	BWA-SW	97.64	44.93	98.31	97.05	98.97	**98.96**
	Bowtie2	97.61	62.92	98.32	91.62	98.96	98.94
	CUSHAW2	97.67	60.67	98.34	**98.12**	98.95	98.95
	RandAL	**97.80**	**93.55**	**98.66**	97.48	**99.08**	98.48

1 All methods performed well at 1% base error rate.

2 With 4% base error rates, the other methods suffered, particularly with shorter reads. The best of them (Bowtie2) got *∼*63% recall at 35 bp. Low recall rate means few reads (out of the total number) were aligned correctly.

3 Our method consistently achieved the highest performance (or among the highest performance) across different read lengths and base error rates. In precision, our method always got the highest, consistently above 97.8%. In recall, even at worst case of 4% base error rate and 35 bp read length, we got *∼*94%.

### Raw running times of top 4 aligners

Theoretically, asymptotic complexity of our method in aligning a read of length *m *is proportional to *m *+ *m*^2^. The worst case complexity of *m*^2 ^is due to edit distance computation. The heuristic for computing edit distance, however, reduces this worst-case complexity significantly in practice. Our testing showed that the running times of other methods, like ours, did not depend much on genome sizes.

Table 3 shows the averaged running times (in seconds) of the 4 aligners in aligning 100,000 reads. Our method suffered with shorter reads, but were the second fastest with longer reads (*≥ *100 bp). It seems that the benefit of randomization becomes more evident with longer reads.

**Table 3:** Average running times of top 4 aligners at different read lengths.

	35 bp	51 bp	76 bp	100 bp	200 bp	400 bp
BWA-SW	8.1	13.4	21.6	30.1	56.9	105.2
Bowtie2	2.8	4.1	5.8	8.1	18.3	41.6
CUSHAW2	4.2	7.8	12.7	19.3	67.8	228.5
RandAL	11.1	12.9	13.6	14.5	26.2	81.6

Bowtie2 was the fastest across the board, but as shown in the previous section, its alignment quality is not as good as our method or CUSHAW2. Compared to ours, CUSHAW2 was significantly slower. Observing running times at different read lengths, we speculate that CUSHAW2 might be much be slower than ours with longer reads.

### Difficulty of alignment in the presence of repeats

Although eukaryotic genomes are expected to be harder to align than bacterial genomes, an examination of performance of the top 4 aligners in Figure 4 reveals that these aligners did not always perform better on bacterial genomes; eukaryotic genomes were not always harder to align. To quantify the degree of difficulty in aligning reads to genomes, we define *repeat density *as a measure of how many repeats a genome has. Since repeats directly affect alignment quality, the notion of repeat density is meant as a quantifiable approximation of genome complexity. More precisely, given a genome  S and one of its length-*k *substrings, *l*, let *f *(*l*) be the number of times *l *occurs in *S*. We define the *k-mer density *of  S given *k *to be

$$D\left(S|k\right)=\frac{\sum_{l\in S,f\left(l\right)\geq 2}f\left(l\right)}{|S|-k+1}$$

*D(S|k)*can be interpreted as the probability that a random read of length *k *is a repeat. The larger D(S|k) is, the more repeats  S has and the harder it is expected for aligners to align k-mer reads to  S. To investigate how much repeat density correlates with the difficulty of aligning short reads to genomes, first, we computed D(S|k) for *k *at each read length 35, 51, 76, 100, 200, and 400. To get a glimpse of its distribution, we show the values of D(S|k) of the bacterial and eukaryotic genomes, for *k*'s equal to these read lengths, in Table 4. Second, for each *k*, we computed the Pearson correlation between D(S|k) of all bacterial and eukaryotic genomes and the performance (precision and recall) of each aligner on aligning reads of length *k *to the genomes.

**Table 4:** Repeat density of genomes, *D*(*S|k*), at various length *k*.

	Genome	Repeat density at various *k*					
		
		35	51	76	100	200	400
Bacteria	*Wolbachia endosymbiont*....	0.181	0.161	0.144	0.134	0.107	0.077
	*Staphylococcus aureus..*.	0.064	0.058	0.053	0.050	0.043	0.036
	*Escherichia coli 042*	0.053	0.044	0.036	0.031	0.023	0.017
	*Pseudomonas aeruginosa *...	0.041	0.037	0.033	0.031	0.026	0.021
	*Streptomyces hygroscopicus *...	0.046	0.042	0.038	0.036	0.031	0.025
	*Sorangium cellulosum *...	0.038	0.030	0.024	0.020	0.015	0.011

Eukaryota	*Debaryomyces hansenii *...	0.036	0.032	0.028	0.025	0.019	0.013
	*Ectocarpus siliculosus *...	0.092	0.073	0.056	0.046	0.030	0.020
	*Schizosaccharomyces pombe *...	0.050	0.047	0.045	0.042	0.036	0.030
	*Caenorhabditis elegans *...	0.138	0.105	0.080	0.066	0.039	0.024
	*Taeniopygia guttata *...	0.129	0.100	0.070	0.050	0.017	0.002
	*Drosophila melanogaster *...	0.068	0.065	0.062	0.060	0.052	0.042

Table 5 shows that repeat density is correlated highly negatively to performance (precision and recall) of all aligners. This means the larger D(S|k) is, the worse any aligner will perform. In other words, D(S|k) is good indicator of alignment difficulty. That said, we also observe that for BWA-SW at small lengths (k = 35, 51), the negative correlation was weakest. This is probably due to BWA-SW trimming short reads.

**Table 5:** Pearson correlation coefficients of repeat density and performance

		k = 35	k = 51	k = 76	k = 100	k = 200	k = 400
Correlation of repeat density and precision	BWA-SW	-0.94	-0.95	-0.96	-0.95	-0.97	-0.95
	Bowtie2	-0.94	-0.95	-0.96	-0.96	-0.97	-0.95
	CUSHAW2	-0.94	-0.94	-0.95	-0.95	-0.95	-0.93
	RandAL	-0.84	-0.83	-0.87	-0.88	-0.93	-0.94

Correlation of repeat density and recall	BWA-SW	-0.64	-0.37	-0.90	-0.95	-0.97	-0.95
	Bowtie2	-0.95	-0.94	-0.96	-0.97	-0.97	-0.96
	CUSHAW2	-0.95	-0.94	-0.95	-0.95	-0.95	-0.93
	RandAL	-0.95	-0.96	-0.97	-0.97	-0.97	-0.96

## Conclusions

We introduced RandAL, a novel randomized approach to aligning reads to reference genomes. We showed that it performed among some of the top aligners that currently exist. Unlike the other aligners, however, RandAL distinctly performs consistently well across a wide range of parameters (read lengths and error rates) across all tested bacterial and eukaryotic genomes. As current sequencing technologies can produce reads in the tested range at low cost [14], our approach promises to work well with these technologies.

Using repeat density as a measure of genome complexity, we showed that this measure correlated highly negatively with alignment quality (precision and recall). This implies that for larger and more complex genomes with many more repeats, these aligners will similarly suffer, as expected.

## Competing interests

The authors declare that they have no competing interests.

## Authors' contributions

VP conceived and designed the algorithm and experiments. NSV, QT, and NN implemented the algorithm. NSV and QT collected data, implemented experiments, carried out evaluation and data analysis. All authors read and approved the manuscript.

## Declarations

Publication charges for this work were funded by NSF grant CCF-1320297 to VP.

This article has been published as part of *BMC Genomics *Volume 15 Supplement 5, 2014: Selected articles from the Third IEEE International Conference on Computational Advances in Bio and Medical Sciences (ICCABS 2013): Genomics. The full contents of the supplement are available online at http://www.biomedcentral.com/bmcgenomics/supplements/15/S5.
